# Supplementary material for: ZMYND11-MBTD1 induces leukemogenesis through hijacking NuA4/TIP60 acetyltransferase complex and a PWWP-mediated chromatin association mechanism
Source: Nat Commun. 2021 Feb 16;12:1045. doi: 10.1038/s41467-021-21357-3 (PMC7886901; doi:10.1038/s41467-021-21357-3)
Supplement: Supplementary file 1 — Supplementary Information [file 41467_2021_21357_MOESM1_ESM.pdf]

Supplementary Fig. 1

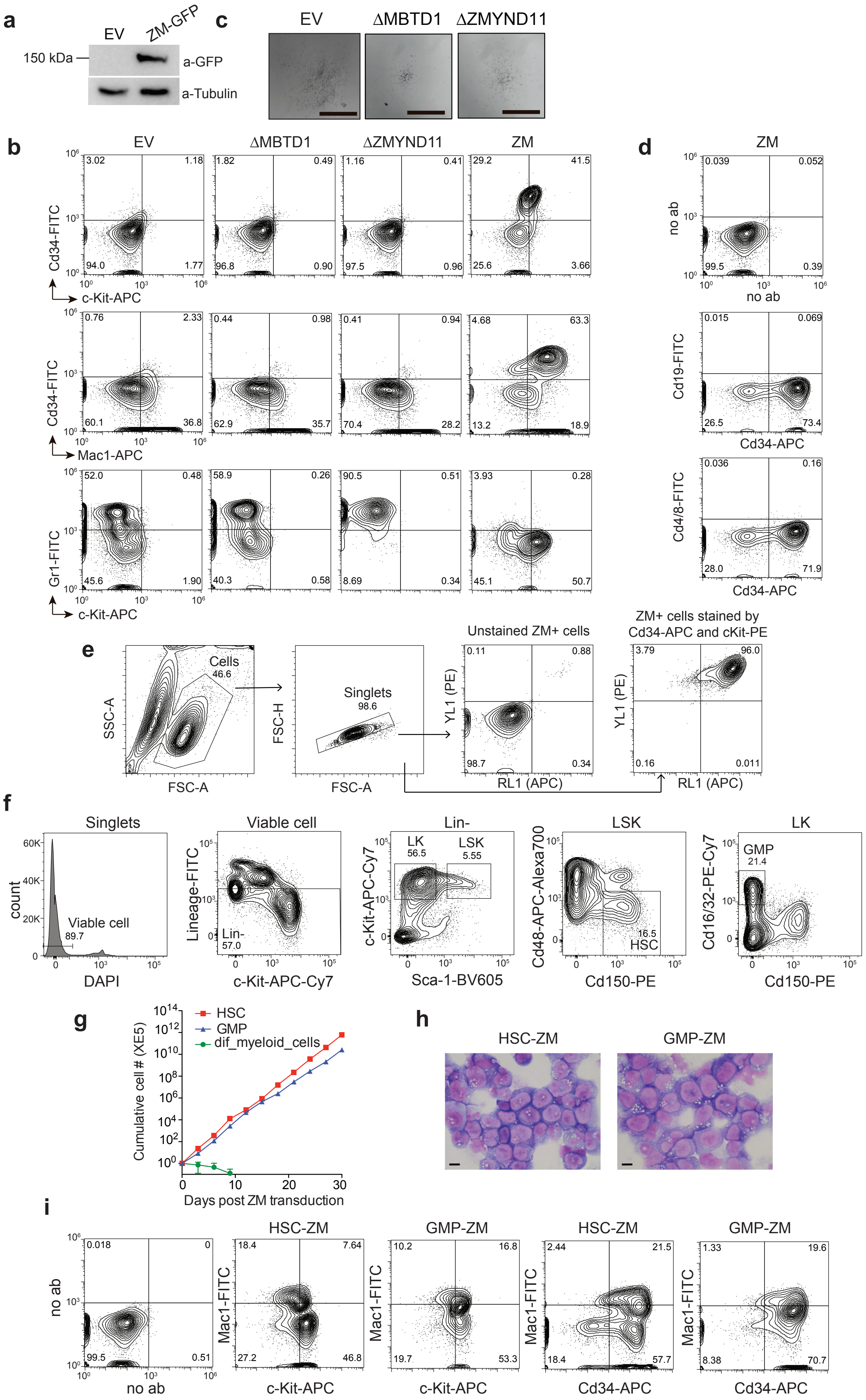

Supplementary Figure 1. ZMYND11-MBTD1 (ZM), a human AML-associated chimeric gene, induces immortalization of murine HSPCs *in vitro*.

a Western blot showing expression of GFP-tagged ZM post-transduction into murine HSPCs.

b FACS of murine HSPC cultures two weeks post-transduction by EV or the indicated ZM (either WT, ∆Z or ∆M).

c Images of representative colonies (at the third replating) seen with HSPCs stably transduced with EV, ∆Z, or ∆M (scale bar = 1 mm).

d FACS of ZM-immortalized murine AML cells.

e FACS gating strategy for analysis of ZM-immortalized murine AML cells. Unstained cells were used as a negative control to determine the gating.

f FACS gating strategy for isolation of murine HSC (hematopoietic stem cell) and GMP (granulocyte-macrophage progenitor).

g Growth curves of ZM-transduced murine HSC, GMP and differentiated myeloid cells (cKit-/Mac1+) in liquid culture (n = 3 biological replicates per group and data is presented as mean ± SD).

h-i Wright-Giemsa staining (panel **h**; scale bar = 10 um) and FACS (panel **i**) of ZM-immortalized murine HSC and GMP.

Supplementary Fig. 2

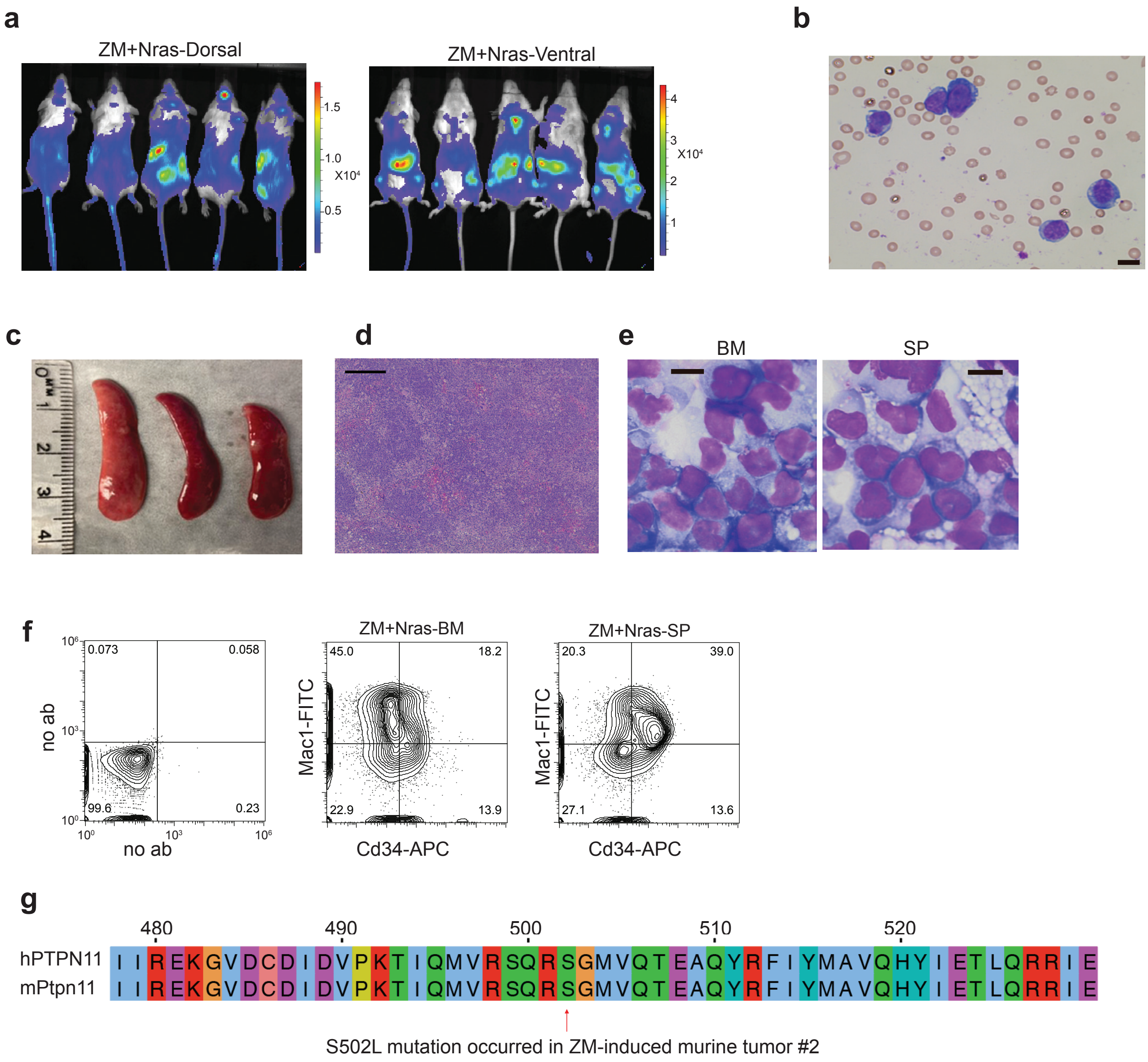

	Normal	Tumor
DNA	TCG	TTG
Protein	Ser	Leu

Supplementary Figure 2. ZM causes AML in mice

a Bioluminescence images of live mice, with dorsal and ventral views shown on the left and right respectively, 18 days post-transplantation of HSPCs co-expressing ZM, $Nras^{G12D}$ and a luciferase reporter.

b-c A representative Wright-Giemsa staining image (panel **b**, scale bar = 10 um) of peripheral blood smear and enlarged spleens (panel **c**) prepared from the terminated mice carrying AML induced by ZM+$Nras^{G12D}$.

d H&E staining of spleen from the mice bearing ZM+$Nras^{G12D}$-induced AML (scale bar = 200 um).

e-f Wright-Giemsa staining (panel **e,** scale bar = 10 um) and FACS (panel **f**) of cells derived from bone marrow (BM) and spleen (SP) in the ZM+$Nras^{G12D}$-engrafted mice that developed full-blown AML.

g Alignment of protein sequences of murine Ptpn11 (NP_001103462.1/1-593) with human PTPN11 (NP_002825.3/1-593). Conserved residues are colored by ClustalX color scheme based on their physico-chemical properties. The table below lists the missense mutation S502L in Ptpn11 gene present in ZM-induced murine tumor #2.

Supplementary Fig. 3

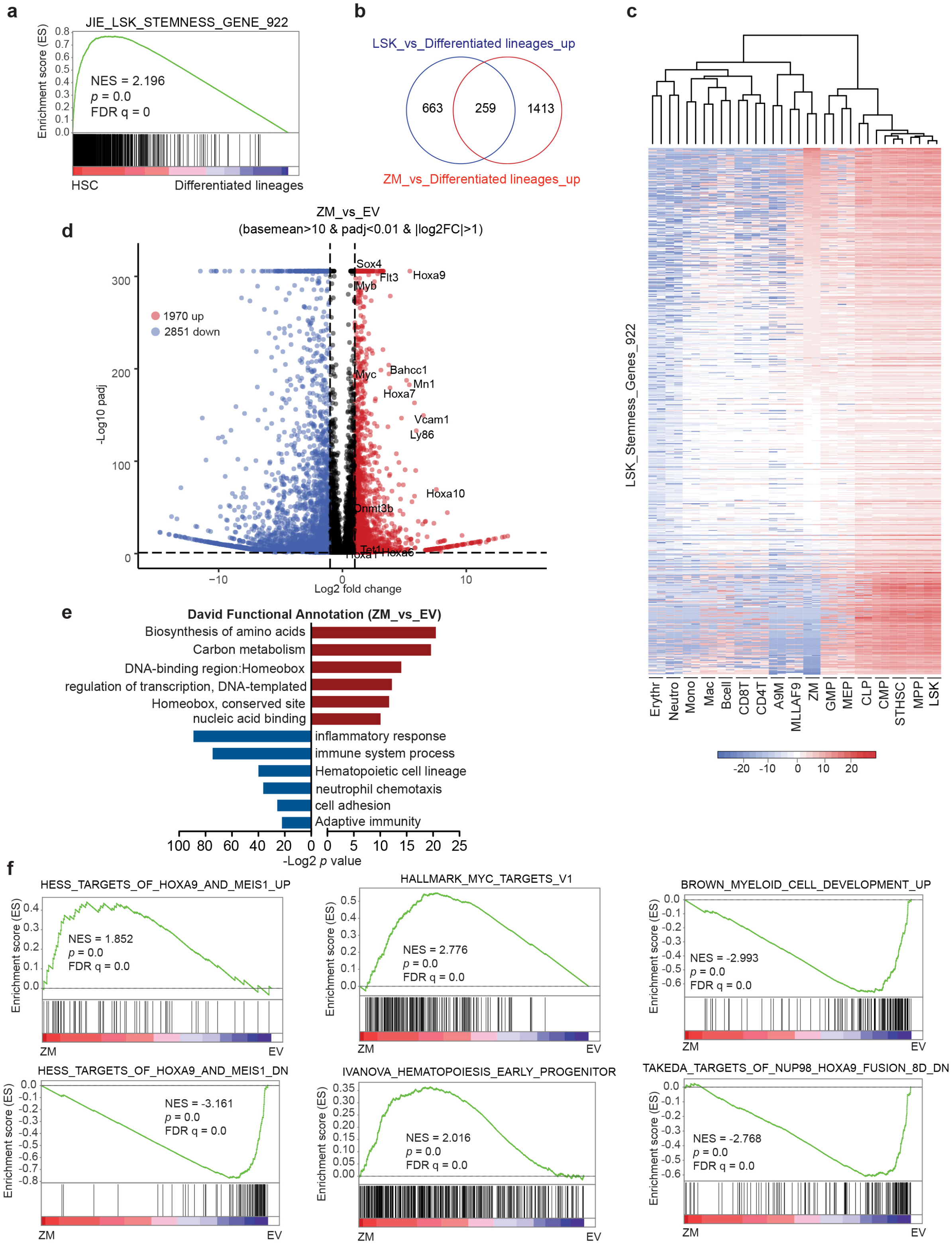

Supplementary Figure 3. ZM enforces aberrant activation of a pro-leukemic stemness gene-expression program including Hoxa, Meis1, Sox4, Myc and Myb.

a GSEA verified enrichment of a LSK “stemness”-related 922-gene signature defined in this work among primitive self-renewing HSPCs (i.e., long-term and short-term hematopoietic stem cell [LT-HSC and ST-HSC] and multipotent progenitor [MPP]), relative to various mature blood cells (B, CD4+ T, CD8+ T, natural killer, macrophage, and granulocyte cells), by using a second publicly available dataset[73]. The *p* value was calculated by an empirical phenotype-based permutation test. The FDR is adjusted for gene set size and multiple hypotheses testing while the *p* value is not.

b Venn diagram shows overlap between LSK “stemness”-related genes and those upregulated in ZM-immortalized AML cells relative to the above-mentioned mature blood cells.

c Hierarchical clustering analysis of the indicated cell types based on the LSK “stemness”-related 922-gene signature. Expression values represent mean-centered log2(Transcripts Per Million [TPM]), and genes were sorted in a descending order based on their expression levels in ZM-immortalized AML cells.

d Volcano plot showing the differentially expressed genes (DEGs) identified in HSPCs transduced with ZM, relative to those with EV. The up- and down-regulated DEGs, defined with the indicated cutoff, are represented in red and blue dots, respectively, with example stemness-related genes highlighted.

e DAVID functional annotation of DEGs identified in HSPCs transduced with ZM, relative to those with EV, with the enriched terms for upregulated (n = 1,970 genes) and

downregulated (n = 2,851 genes) DEGs indicated by red and blue bars, respectively. The *p* value was calculated by Fisher's Exact test.

f GSEA analyses showed enrichments of the indicated gene signatures in either ZM or EV-transduced HSPCs. The *p* value (n = 15,282 genes per group) was calculated by an empirical phenotype-based permutation test. The FDR is adjusted for gene set size and multiple hypotheses testing while the *p* value is not.

Supplementary Fig. 4

a

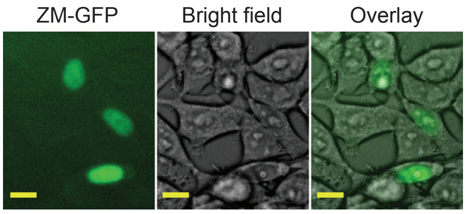

b

	Total # of raw reads	Total # of raw reads after alignment	Total # of aligned reads with PCR-duplicated ones removed
**ZM fusion GFP-tag ChIP**	29,401,683	27,225,778	24,276,651
**Input for ZM fusion (GFP ChIP)**	23,531,123	21,011,746	18,668,349
**ZM fusion HA-tag ChIP**	31,508,735	28,965,070	25,546,155
**Input for ZM fusion (HA ChIP)**	26,466,377	23,593,650	20,751,057
**ZM H3K27me3 ChIP**	56,712,756	52,661,623	47,649,326
**ZM H3K36me3** ChIP	62,575,395	59,541,013	53,995,811
**ZM H3K27ac ChIP**	30,967,066	29,582,385	26,923,029
**ZM H4ac ChIP**	33,080,941	30,863,802	27,679,374
**Input for ZM histone ChIP**	37,336,117	34,040,518	29,818,649
**ZM Tip60 ChIP (combined replicates)**	45,943,075	41,765,138	36,811,952
**Input for ZM Tip60 ChIP**	26,383,904	24,154,198	21,346,057
**ZM Brd4 ChIP**	56,560,794	51,809,672	45,236,732
**Input for Brd4 ChIP**	21,018,190	19,145,377	16,794,007

c

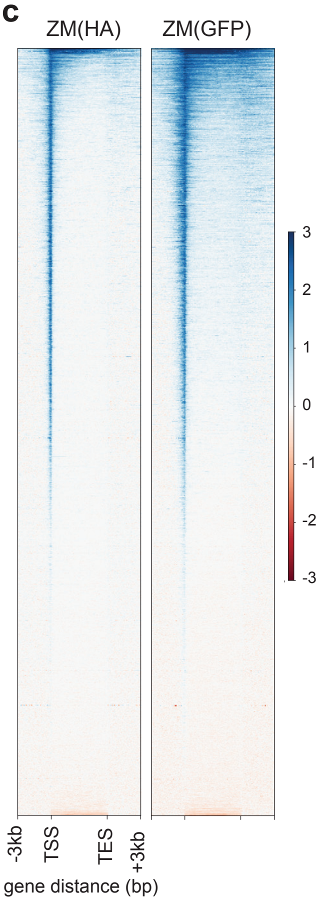

d

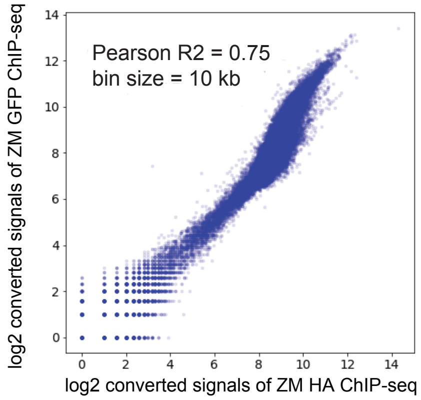

f

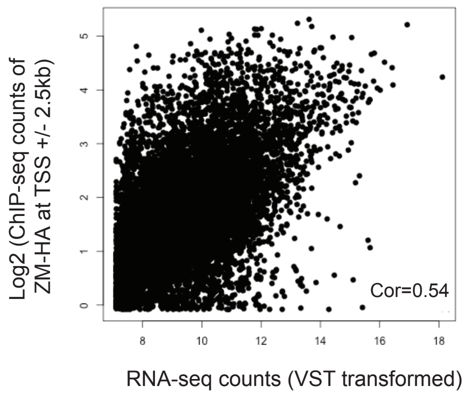

g

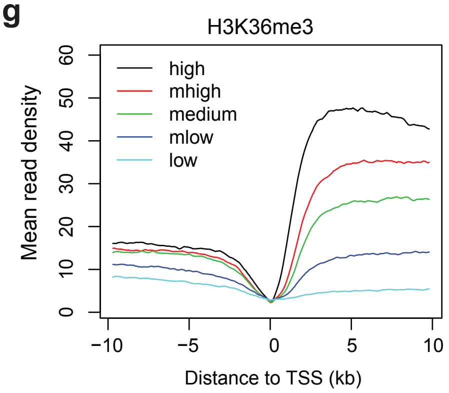

h

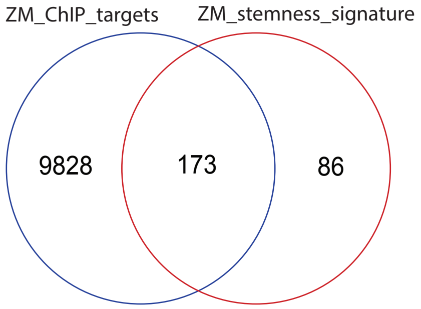

e

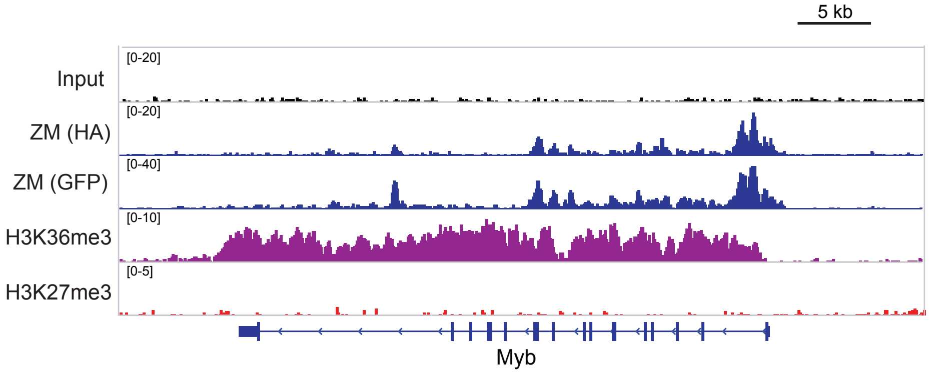

Supplementary Figure 4. Integrated ChIP-seq and RNA-seq analyses revealed a positive correlation between ZM binding and proto-oncogene activation.

a Fluorescent microscopy image showing that the GFP-tagged ZM displays an exclusive nuclear localization pattern in 293T stable cells (scale bar = 10 um).

b Summary of sequencing depth and total number of aligned reads after removal of PCR-duplicated ones for the indicated ChIP-seq experiments.

c Heatmaps showing overlap of ZM HA and GFP ChIP-seq signals along the transcription unit (from TSS to TES with 3 kb extension upstream of TSS and downstream of TES). All genes in the genome are included and sorted in a descending order based on the mean value per region.

d Scatterplot showing a strong correlation between two different ZM ChIP-seq signals using either HA or GFP antibodies and HSPCs immortalized by either 3HA-3Flag or GFP-tagged ZM. The correlation coefficient (R^2) was calculated by Pearson's correlation.

e ChIP-seq profiles (RPGC and input-normalized) of ZM, H3K36me3, and H3K27me3 at the Myb gene locus.

f Scatterplot showing strong positive correlation of ZM ChIP tag densities at promoter-proximal regions with its target gene mRNA abundances. RNA-seq read counts were normalized by variance-stabilizing transformation (VST).

g Positive correlation between H3K36me3 ChIP-seq signal densities and target mRNA abundancies as revealed by RNA-seq. Genes were equally divided into 5 groups from high, medium high ("mhigh"), medium, medium low ("mlow"), to low expression.

h Venn diagram shows that a majority of the ZM-activated stemness genes are directly bound by ZM as revealed by ChIP-seq.

Supplementary Fig. 5

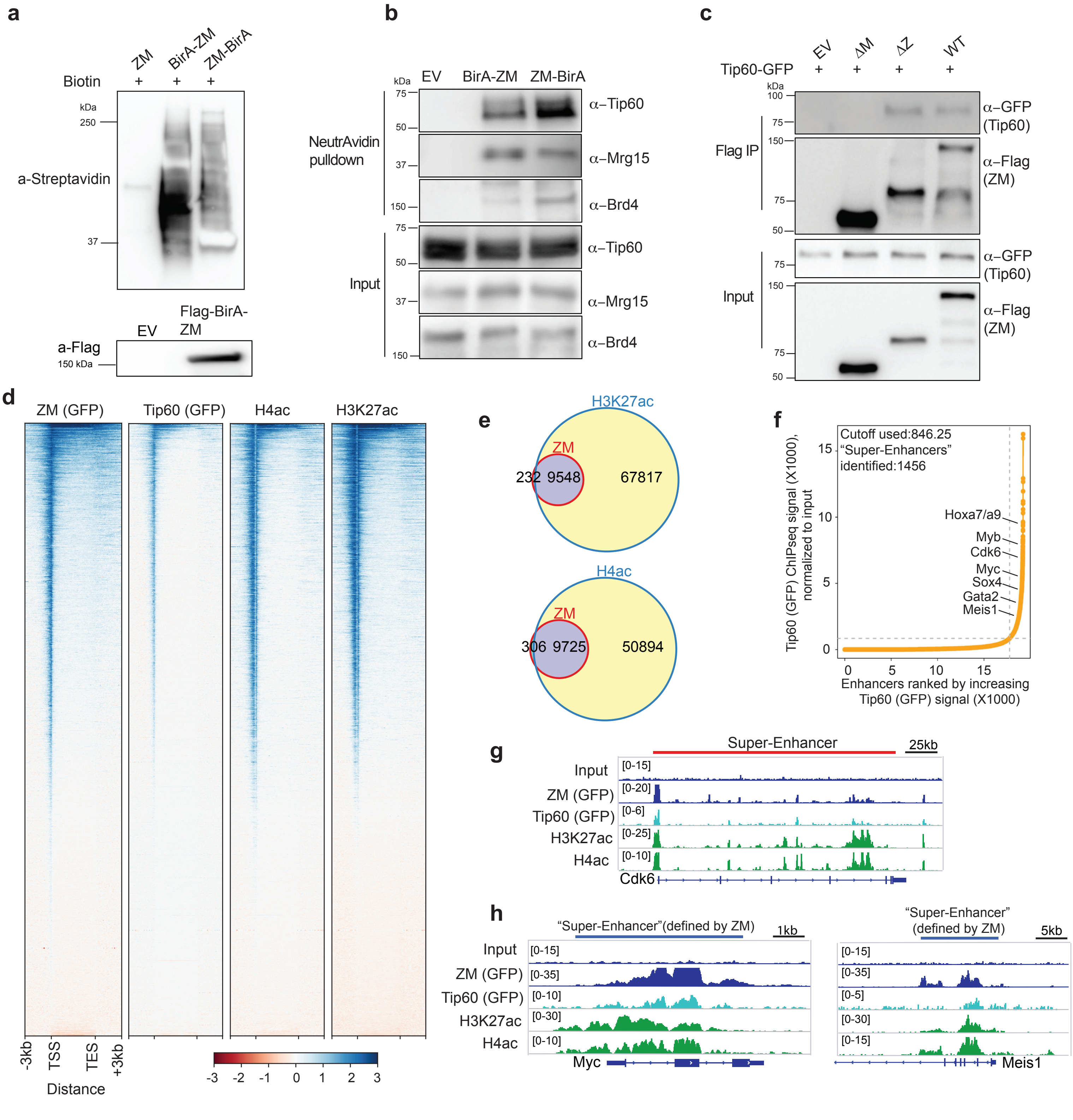

Supplementary Figure 5. ZM interacts with NuA4/Tip60 complex, establishing "super-enhancers" at target proto-oncogenes.

a-b Western blots detecting the biotinylated ZM-interacting proteins after pulldown with the NeutrAvidin agarose, by using antibodies of streptavidin (panel **a**; bottom shows immunoblot for Flag-tagged BirA-ZM fusion used in BioID) or the indicated protein (panel **b**).

c Co-IP for assessing the interaction between GFP-tagged Tip60 and 3XHA-3XFlag-tagged ZM, either WT or with fusion partner deleted (ΔZ or ΔM), after their co-expression in 293T cells. Empty vector (EV) serves as control.

d Heatmaps showing overlap of the indicated ChIP-seq signal densities along the transcription unit (from TSS to TES with 3 kb extension upstream of TSS and downstream of TES); the whole-genome genes are included and sorted in descending order based on the mean value per region.

e Venn diagram showing the number of overlapped peaks between the indicated ChIP-seqs.

f Hockey-stick plot shows distribution of input-normalized Tip60 ChIP-seq signals across all enhancers annotated by H3K27ac peaks. Representative super-enhancers-associated pro-leukemic genes are indicated.

g-h ChIP-seq profiles (RPGC and input-normalized) of ZM, Tip60, H3K27ac, and H4ac at the indicated gene loci. Super-enhancers defined by H3K27ac or ZM are depicted with a red bar and blue bar on top, respectively.

Supplementary Fig. 6

a

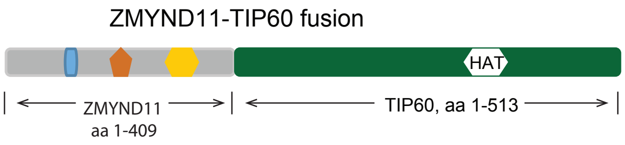

b

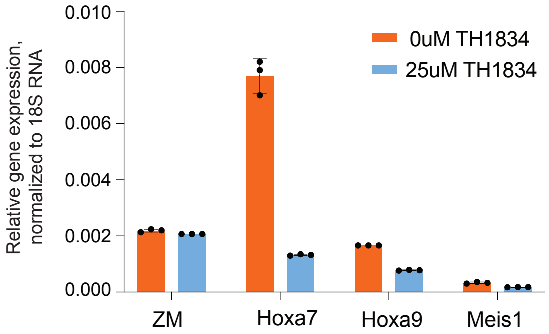

Supplementary Figure 6. Recruitment of NuA4/Tip60 complex is essential for ZM-induced target gene activation and leukemic transformation.

a Scheme showing domain structure of an artificial ZMYND1-TIP60 fusion, which is created by replacing the MBDT1 fusion segment of ZM with a full-length cDNA of human TIP60. HAT, histone acetyltransferase domain.

b RT-qPCR analysis of the indicated genes in ZM-transformed AML cells, either mock treated or treated with 25 uM of TH1834 for six days. qPCR signals from three independent experiments were normalized to internal control 18S RNA and presented as mean ± SD.

Supplementary Fig. 7

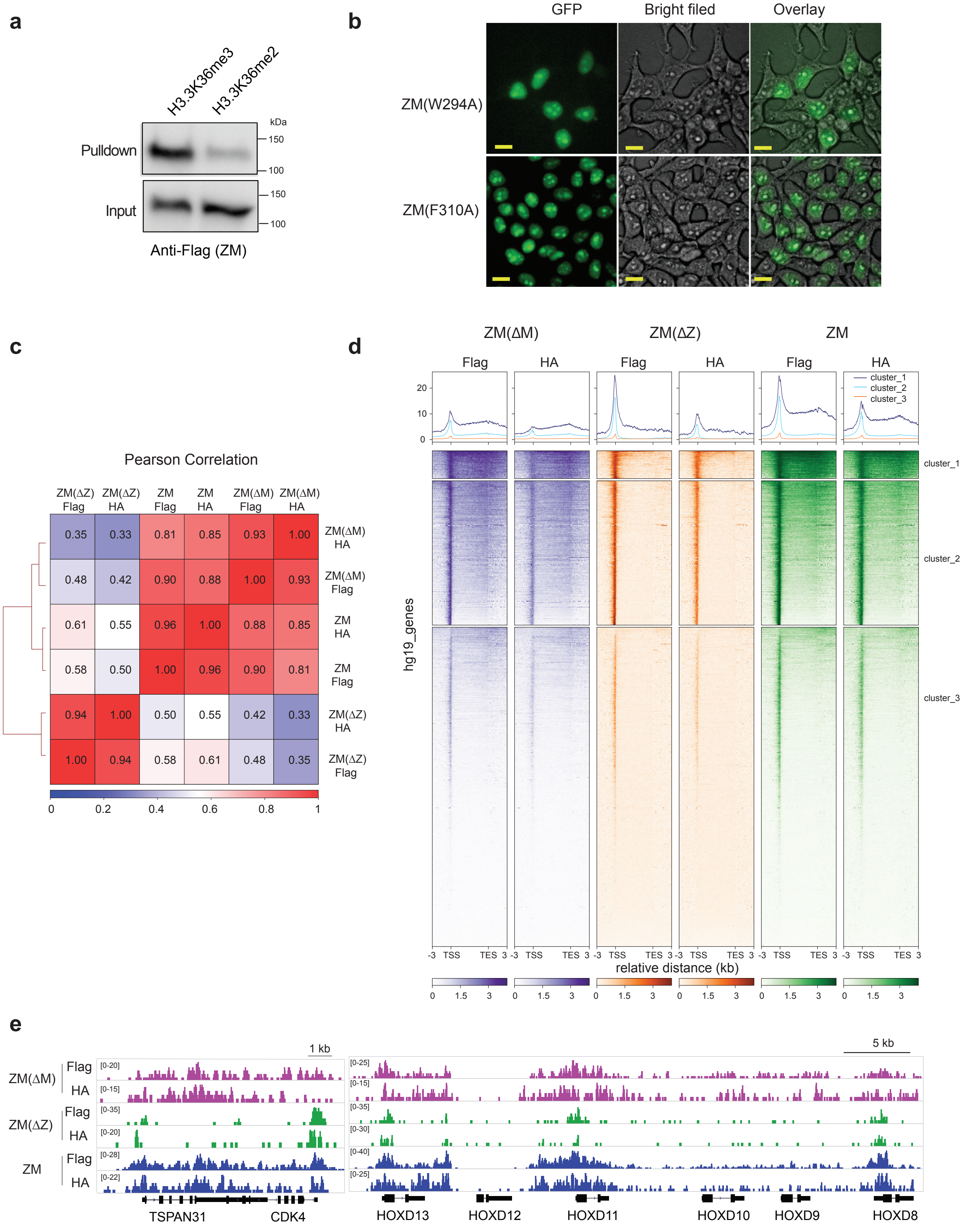

Supplementary Figure 7. An H3.3K36me3-binding PWWP motif is essential for chromatin association of ZM, as well as ZM-enforced proto-oncogene activation and leukemic transformation.

a Pulldown assays using the histone H3.3 peptides modified by either K36me2 or K36me3 and total protein extracts of 293T cells with stable expression of 3XHA-3XFlag-tagged ZM.

b Fluorescent microscopy shows that the indicated GFP-tagged ZM mutants (W294A and F310A) display a nuclear distribution pattern in 293T stable cells. Scale bar = 10 um.

c Hierarchical clustering of the indicated CUT&RUN datasets based on similarity, with the pairwise Pearson correlation coefficients labeled in table and depicted by varying color intensities.

d Average signal profiles and clustered heatmaps displaying CUT&RUN signals (detected using either Flag or HA antibody) for 3XHA-3XFlag-tagged ZMΔM, ZMΔZ, and ZM over all hg19 genes in 293T cells.

e IGV tracks showing the distribution of ZMΔM, ZMΔZ, and ZM CUT&RUN signals (detected using either Flag or HA antibody) at the indicated gene in 293T cells.

Supplementary Fig. 8

a

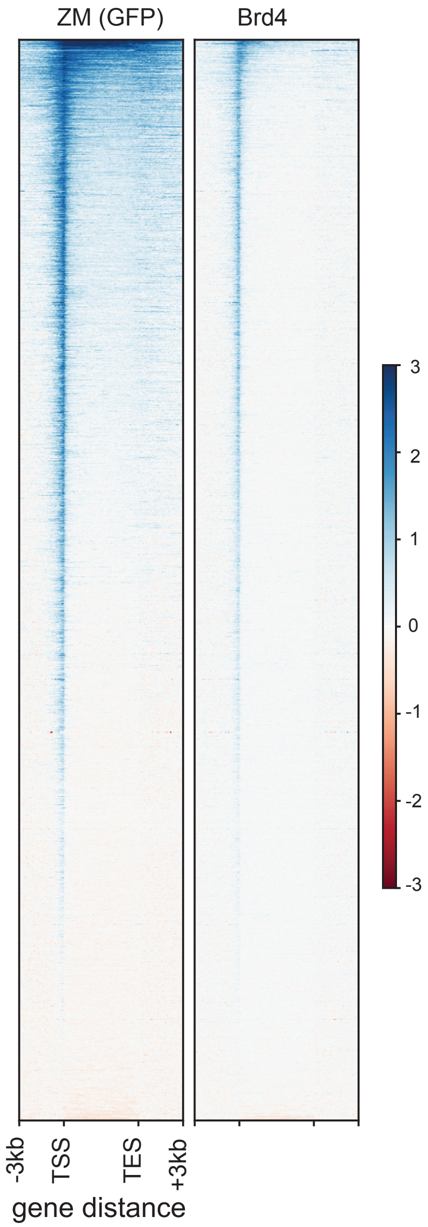

b

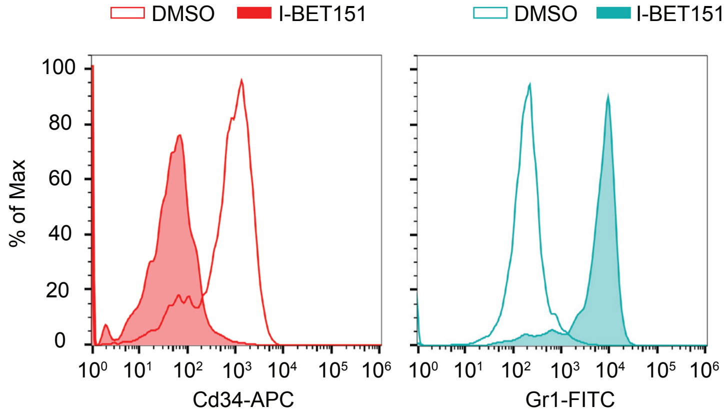

c

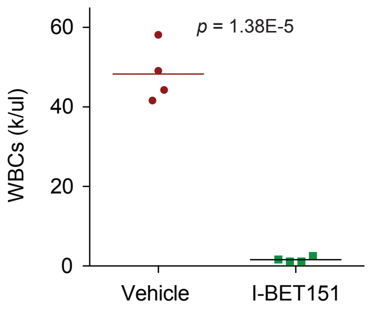

Supplementary Figure 8. ZM-induced AML is sensitive to Brd4 blockade.

a Heatmaps showing overlap of the indicated ChIP-seq signal densities along the transcription unit (from TSS to TES with 3 kb extension upstream of TSS and downstream of TES); the whole-genome genes are included and sorted in descending order based on the mean value per region.

b FACS analysis for Cd34 and Gr-1 surface marker expression in the ZM-transformed AML cells post-treatment with DMSO or I-BET151 (0.25 uM) for four days.

c Counts of WBCs in the peripheral blood in the indicated cohort (n = 4 mice per group) at the end point of mock-treated ones. The *p* value was calculated by two-sided Student's t test.
