# Supplementary material for: ZMYND11-MBTD1 induces leukemogenesis through hijacking NuA4/TIP60 acetyltransferase complex and a PWWP-mediated chromatin association mechanism
Source: Nat Commun. 2021 Feb 16;12:1045. doi: 10.1038/s41467-021-21357-3 (PMC7886901; doi:10.1038/s41467-021-21357-3)
Supplement: Supplementary file 3 — Description of Additional Supplementary Files [file 41467_2021_21357_MOESM3_ESM.pdf]

Description of Additional Supplementary Files

Supplementary Data 1
Nonsynonymous mutations identified by mWES in ZM alone-induced primary murine leukemia samples.

Supplementary Data 2
RNA-seq based transcriptome profiling revealed that ZM-transformed murine AML cells express a subset (15%) of LSK 'stemness'-related signature genes.

Supplementary Data 3
RNA-seq identified genes, including a LSK 'stemness' gene signature, significantly upregulated in the ZM- or MLL-AF9-transformed murine AML cells, relative to those transformed by co-expression of Hoxa9 plus Meis1 (A9M).

Supplementary Data 4
Integrated ChIP-seq and RNA-seq analyses revealed genes directly activated by ZM during leukemic transformation.

Supplementary Data 5
ZM-interacting proteins identified by BioID using murine HSPCs stably expressing ZM fused with a BirA domain at either its N- or C-terminus.

Supplementary Data 6
Primer sequences used in this study.
