# Supplementary material for: ZMYND11-MBTD1 induces leukemogenesis through hijacking NuA4/TIP60 acetyltransferase complex and a PWWP-mediated chromatin association mechanism
Source: Nat Commun. 2021 Feb 16;12:1045. doi: 10.1038/s41467-021-21357-3 (PMC7886901; doi:10.1038/s41467-021-21357-3)
Supplement: Supplementary file 10 — Reporting Summary [file 41467_2021_21357_MOESM10_ESM.pdf]

nature research

Corresponding author(s): Gang Greg Wang

Last updated by author(s): Jan 20, 2021

Reporting Summary

Nature Research wishes to improve the reproducibility of the work that we publish. This form provides structure for consistency and transparency in reporting. For further information on Nature Research policies, see our Editorial Policies and the Editorial Policy Checklist.

Statistics

For all statistical analyses, confirm that the following items are present in the figure legend, table legend, main text, or Methods section.

n/a	Confirmed	
☐	☒	The exact sample size (n) for each experimental group/condition, given as a discrete number and unit of measurement
☐	☒	A statement on whether measurements were taken from distinct samples or whether the same sample was measured repeatedly
☐	☒	The statistical test(s) used AND whether they are one- or two-sided<br>*Only common tests should be described solely by name; describe more complex techniques in the Methods section.*
☒	☐	A description of all covariates tested
☒	☐	A description of any assumptions or corrections, such as tests of normality and adjustment for multiple comparisons
☐	☒	A full description of the statistical parameters including central tendency (e.g. means) or other basic estimates (e.g. regression coefficient) AND variation (e.g. standard deviation) or associated estimates of uncertainty (e.g. confidence intervals)
☐	☒	For null hypothesis testing, the test statistic (e.g. F, t, r) with confidence intervals, effect sizes, degrees of freedom and P value noted<br>*Give P values as exact values whenever suitable.*
☒	☐	For Bayesian analysis, information on the choice of priors and Markov chain Monte Carlo settings
☒	☐	For hierarchical and complex designs, identification of the appropriate level for tests and full reporting of outcomes
☐	☒	Estimates of effect sizes (e.g. Cohen's d, Pearson's r), indicating how they were calculated

Our web collection on statistics for biologists contains articles on many of the points above.

Software and code

Policy information about availability of computer code

Data collection	NextSeq System Suite v2.2.0, Attune™ NxT Software v2.7
Data analysis	RNA-seq data analysis:<br>The fastq files were aligned to the mm10 mouse genome (GRCm38.p4) using STAR v2.4.2 with the following parameters: --outSAMtype BAM Unsorted --quantMode TranscriptomeSAM. Transcript abundance for each sample was estimated with salmon v0.1.19 to quantify the transcriptome defined by Gencode gene annotation. Gene level counts were summed across isoforms and genes with low expression (all samples had less than 10 reads) were removed before downstream analyses. DESeq2 were used to test for differentially expressed genes between different samples.<br><br>ChIP-seq data analysis:<br>Trim galore version 0.6.1 was used for adapter trimming and quality filter for all reads. STAR v2.7.1a was used to align the reads to the mouse genome (mm9). Samtools (v1.9), Picard MarkDuplicates funtion (ver 2.20.4), and bedtools (v2.28.0) were used to remove the non-primary alignment, PCR duplicates, or blacklist regions from aligned data. MACS2(v2.1.1) was used for peak calling. Deeptools (v3.3.0) was used to make bigwig files, heatmaps, and averaged plottings of ChIP-seq signal. These bigwig files were visualized using IGV v2.5.3. Custom scripts of R (v3.5.0) or Python(v3.6) were used for some statistical analysis.<br><br>CUT&RUN Data Analysis<br>Raw sequencing reads were first trimmed by Trim Galore version 0.6.1 to remove low-quality and adaptor bases, and then aligned to hg19 using STAR v2.7.1a. After mapping reads to the genome, only primary alignments were extracted followed by removal of duplicate reads using the Picard MarkDuplicates tool (ver 2.20.4). The bigwig coverage file was generated from BAM alignment file using the deeptools (v3.3.0) bamCoverage function and the number of reads per bin was normalized to 1x genome coverage (reads per genome coverage, RPGC). The clustered correlation heatmap was made using the deepTools plotCorrelation function and correlation coefficients were computed by Pearson method.

FACS Data Analysis
FACS data were analyzed by FlowJo 10.7.1. Specifically, the cell populations were gated based on FSC-A and SSC-A; singlets were gated based on FSC-H and FSC-A; negative populations were gated based on unstained controls.

For manuscripts utilizing custom algorithms or software that are central to the research but not yet described in published literature, software must be made available to editors and reviewers. We strongly encourage code deposition in a community repository (e.g. GitHub). See the Nature Research guidelines for submitting code & software for further information.

Data

Policy information about availability of data

All manuscripts must include a data availability statement. This statement should provide the following information, where applicable:
- Accession codes, unique identifiers, or web links for publicly available datasets
- A list of figures that have associated raw data
- A description of any restrictions on data availability

RNA-seq, ChIP-seq and CUT&RUN datasets related to this work have been deposited in the NCBI GEO under accession number GSE150428. The mWES data are available in the NCBI SRA under the accession code PRJNA693299. The Mass Spectrometry proteomics data have been deposited to the ProteomeXchange Consortium via the PRIDE partner repository with the dataset identifier PXD023702. The source data underlying Figures 1r, 5b-5c, 5h, 6b, 6e-6g and Supplementary Figures 5a-5c, 7a are provided in the Source Data file. All the other data supporting the findings of this study are available within the article and its Supplementary Information files and from the corresponding author upon reasonable request.

Field-specific reporting

Please select the one below that is the best fit for your research. If you are not sure, read the appropriate sections before making your selection.

☒ Life sciences ☐ Behavioural & social sciences ☐ Ecological, evolutionary & environmental sciences

For a reference copy of the document with all sections, see nature.com/documents/nr-reporting-summary-flat.pdf

Life sciences study design

All studies must disclose on these points even when the disclosure is negative.

Sample size	No statistical measures were used to determine sample size. Sample sizes were estimated based on prior experiments in our laboratory and previously published data.
Data exclusions	No data were excluded from the analyses.
Replication	All experimental findings could reliably be reproduced independently two to three times.
Randomization	Mice were randomly allocated into experimental groups by mouse core facility at UNC-CH.
Blinding	Investigators were blinded to group allocation during data collection. Blinded data analysis was carried out as well, except when data were analyzed by pre-defined, well-accepted cut-offs and not easily subjected to investigator bias.

Reporting for specific materials, systems and methods

We require information from authors about some types of materials, experimental systems and methods used in many studies. Here, indicate whether each material, system or method listed is relevant to your study. If you are not sure if a list item applies to your research, read the appropriate section before selecting a response.

Materials & experimental systems

n/a	Involved in the study	
☐	☒	Antibodies
☐	☒	Eukaryotic cell lines
☒	☐	Palaeontology and archaeology
☐	☒	Animals and other organisms
☒	☐	Human research participants
☒	☐	Clinical data
☒	☐	Dual use research of concern

Methods

n/a	Involved in the study	
☐	☒	ChIP-seq
☐	☒	Flow cytometry
☒	☐	MRI-based neuroimaging

April 2020

Antibodies

Antibodies used	Antibodies used for western blots include anti-Flag (M2)-HRP (Sigma, A8592), anti-H3 (CST, 9715), anti-GAPDH (CST, 2118), anti-β-Tubulin (CST, 2146), Streptavidin-HRP (CST, 3999) and anti-GFP (CST, 2956).<br><br>Antibodies used for FACS include: c-KitAPC (Invitrogen, 17-1172-82), c-KitFITC (eBioscience,11-1171-85), Cd34APC (eBioscience, 50-0341-82), Cd34FITC (BD, 560238), Mac1APC (BD, 557686), Mac1FITC (eBioscience, 11-0112-85), Gr1FITC (eBioscience, 11-5931-85), Cd4FITC (eBioscience,11-0042-82), Cd8aFITC (eBioscience,11-0081-82), Cd19FITC (eBioscience,11-0193-82).<br><br>The following antibodies were used in ChIP or CUT&RUN assays: anti-Flag (Sigma, F1804), anti-HA (Abcam, ab9110), anti-GFP (Abcam, ab290), anti-H3K36me3 (Abcam, ab9050), anti-H3K27ac (Abcam, ab4729), anti-H3K27me3 (Millipore, 07-449), anti-H4ac (Millipore, 06-866), anti-BRD4 (Bethyl, A301-985A100), anti-Tip60 (Santa Cruz, sc-166323).
Validation	All the antibodies used were validated by the manufactures and the validating data was provided on their websites. Furthermore, we also used positive and negative controls to further validate the antibody specificity by western blotting or immunofluorescence . For histone antibodies, specificity was also extensively examined by many independent investigators in addition to the vendor (refer to http://www.histoneantibodies.com/).

Eukaryotic cell lines

Policy information about cell lines

Cell line source(s)	HEK293T (ATCC #CRL-3216), murine AML cell lines were generated by retroviral transduction of ZM, or A9M, or MLLAF9 into mouse bone marrow-derived HSPCs.
Authentication	Authentication of HEK293T, including parental and their derived lines, was ensured by the Tissue Culture Facility (TCF) affiliated to UNC Lineberger Comprehensive Cancer Center with the genetic signature profiling and fingerprinting analysis. Murine AML cell lines were authenticated by validating the expression of the transduced protein via western blot and RT-qPCR.
Mycoplasma contamination	Cell lines were tested negative for mycoplasma contamination. Every month, a routine examination of cell lines in culture for any possible mycoplasma contamination was performed using commercially available detection kits (Lonza).
Commonly misidentified lines<br>(See ICLAC register)	no commonly misidentified cell lines were used in the study.

Animals and other organisms

Policy information about studies involving animals; ARRIVE guidelines recommended for reporting animal research

Laboratory animals	Female BALB/c and C57BL/6 mice (age of 8 weeks) were purchased from Jackson Labs and maintained by Animal Studies Core, the UNC Lineberger Comprehensive Cancer Center.
Wild animals	no wild animals were used in the study.
Field-collected samples	no field-collected samples were used in the study.
Ethics oversight	All animal experiments were approved by and performed in accord with the guidelines of the Institutional Animal Care and Use Committee at the University of North Carolina (UNC).

Note that full information on the approval of the study protocol must also be provided in the manuscript.

ChIP-seq

Data deposition

- [x] Confirm that both raw and final processed data have been deposited in a public database such as GEO.
- [x] Confirm that you have deposited or provided access to graph files (e.g. BED files) for the called peaks.

Data access links<br>*May remain private before publication.*	https://www.ncbi.nlm.nih.gov/geo/query/acc.cgi?acc=GSE150428
Files in database submission	ZM_fusion_HA_ChIP_input.fastq.gz<br>ZM_fusion_HA_ChIP.fastq.gz<br>ZM_fusion_GFP_ChIP_input.fastq.gz<br>ZM_fusion_GFP_ChIP.fastq.gz<br>ZM_histoneChIP_input.fastq.gz<br>ZM_H3K36me3_ChIP.fastq.gz<br>ZM_H3K27me3_ChIP.fastq.gz

ZM_H3K27ac_ChIP.fastq.gz
ZM_H4ac_ChIP.fastq.gz
ZM_Tip60_GFP_ChIP_input.fastq.gz
ZM_Tip60_GFP_ChIP_rep1.fastq.gz
ZM_Tip60_GFP_ChIP_rep2.fastq.gz
ZM_Brd4_ChIP_input.fastq.gz
ZM_Brd4_ChIP.fastq.gz
ZM_fusion_HA_ChIP_input.bw
ZM_fusion_HA_ChIP_normalized.bw
ZM_fusion_GFP_ChIP_input.bw
ZM_fusion_GFP_ChIP_normalized.bw
ZM_histoneChIP_input.bw
ZM_H3K36me3_ChIP_normalized.bw
ZM_H3K27me3_ChIP_normalized.bw
ZM_H3K27ac_ChIP_normalized.bw
ZM_H4ac_ChIP_normalized.bw
ZM_Tip60_GFP_ChIP_input.bw
ZM_Tip60_GFP_ChIP_combined_normalized.bw
ZM_Brd4_ChIP_input.bw
ZM_Brd4_ChIP_normalized.bw

Genome browser session (e.g. UCSC): no longer applicable

Methodology

Replicates	Two biological replicates for Tip60 GFP ChIP-Seq.
Sequencing depth	ChIP-seq depth was described in the supplemental document (listed in a table).
Antibodies	Described in the above used antibodies filed and GEO link.
Peak calling parameters	MACS2 with the parameter: --nomodel --pvalue 1e-05 --extsize 250
Data quality	ChIP-seq reads were aligned to mouse genome build NCBI37/mm9 using STAR version 2.7.1a after quality trimming. Non-primary alignment and PCR duplicates were then removed by samtools.
Software	STAR (v2.7.1a), MACS(v2.1.1), DeepTools(v3.3.0)

Flow Cytometry

Plots

Confirm that:

- [x] The axis labels state the marker and fluorochrome used (e.g. CD4-FITC).
- [x] The axis scales are clearly visible. Include numbers along axes only for bottom left plot of group (a 'group' is an analysis of identical markers).
- [x] All plots are contour plots with outliers or pseudocolor plots.
- [x] A numerical value for number of cells or percentage (with statistics) is provided.

Methodology

Sample preparation	Cells were washed and suspended in FACS stain buffer (5% FBS in PBS) to a final density of 1E7 cells/ml, followed by incubation with fluorescently (FITC/APC/PE/BV605/AF700/PE-Cy7) conjugated primary antibodies at 1:100 dilution for 30 min on ice, washing with FACS stain buffer before Flow Cytometry analysis.
Instrument	Thermo Fisher Attune NxT and Becton Dickinson FACSAria III (UNC Flow Cytometry Core Facility)
Software	FlowJo software
Cell population abundance	We did two rounds of sorting for mouse HSC and GMP, the purity after second sorting was > 99%.
Gating strategy	Unstained control was used to set negative gate.

- [x] Tick this box to confirm that a figure exemplifying the gating strategy is provided in the Supplementary Information.
